# Design and Usage of the HeartCycle Education and Coaching Program for Patients With Heart Failure

**DOI:** 10.2196/resprot.3411

**Published:** 2014-12-11

**Authors:** Wim Stut, Carolyn Deighan, Wendy Armitage, Michelle Clark, John G Cleland, Tiny Jaarsma

**Affiliations:** ^1^Philips ResearchEindhovenNetherlands; ^2^NHS LothianThe Heart Manual DepartmentEdinburghUnited Kingdom; ^3^National Heart & Lung InstituteRoyal Brompton & Harefield HospitalsImperial CollegeLondonUnited Kingdom; ^4^Linköping UniversityFaculty of Health SciencesLinköpingSweden

**Keywords:** e-counseling, heart failure, lifestyle, patient adherence, self-care, telehealth

## Abstract

**Background:**

Heart failure (HF) is common, and it is associated with high rates of hospital readmission and mortality. It is generally assumed that appropriate self-care can improve outcomes in patients with HF, but patient adherence to many self-care behaviors is poor.

**Objective:**

The objective of our study was to develop and test an intervention to increase self-care in patients with HF using a novel, online, automated education and coaching program.

**Methods:**

The online automated program was developed using a well-established, face-to-face, home-based cardiac rehabilitation approach. Education is tailored to the behaviors and knowledge of the individual patient, and the system supports patients in adopting self-care behaviors. Patients are guided through a goal-setting process that they conduct at their own pace through the support of the system, and they record their progress in an electronic diary such that the system can provide appropriate feedback. Only in challenging situations do HF nurses intervene to offer help. The program was evaluated in the HeartCycle study, a multicenter, observational trial with randomized components in which researchers investigated the ability of a third-generation telehealth system to enhance the management of patients with HF who had a recent (<60 days) admission to the hospital for symptoms or signs of HF (either new onset or recurrent) or were outpatients with persistent New York Heart Association (NYHA) functional class III/IV symptoms despite treatment with diuretic agents. The patients were enrolled from January 2012 through February 2013 at 3 hospital sites within the United Kingdom, Germany, and Spain.

**Results:**

Of 123 patients enrolled (mean age 66 years (SD 12), 66% NYHA III, 79% men), 50 patients (41%) reported that they were not physically active, 56 patients (46%) did not follow a low-salt diet, 6 patients (5%) did not restrict their fluid intake, and 6 patients (5%) did not take their medication as prescribed. About 80% of the patients who started the coaching program for physical activity and low-salt diet became adherent by achieving their personal goals for 2 consecutive weeks. After becoming adherent, 61% continued physical activity coaching, but only 36% continued low-salt diet coaching.

**Conclusions:**

The HeartCycle education and coaching program helped most nonadherent patients with HF to adopt recommended self-care behaviors. Automated coaching worked well for most patients who started the coaching program, and many patients who achieved their goals continued to use the program. For many patients who did not engage in the automated coaching program, their choice was appropriate rather than a failure of the program.

## Introduction

Heart failure (HF) is common and associated with high rates of disability, hospital readmission, and mortality, all of which can be improved by high-quality care [1]. However, management is complex, and health care resources are limited. Enabling and empowering patients, as well as their informal caregivers, to be active participants in the delivery of care (ie, self-care) could improve the quality of care in an efficient and affordable manner [2]. Self-care behaviors of patients with HF include taking medication as prescribed, engaging in physical activity and exercise, eating a low-salt diet, restricting fluid intake, and monitoring signs and symptoms [1]. Patients with HF who report effective self-care have lower mortality and readmission rates than those who report poor self-care [3]. Therefore, engaging patients in self-care is advantageous for both health care systems and patients themselves.

In order for patients to be able to engage in optimal self-care, they and their caregivers are educated about HF and how they may contribute to their own health care [4]. Education may take place during hospitalization, at the outpatient clinic, in the community, or at home. It is typically delivered by health care professionals in face-to-face sessions and supported by booklets or digital media (eg, CD-ROM [5], websites [6], telehealth systems [7], and tablet computers [8]).

Unfortunately, adherence to most self-care behaviors is remarkably low among patients with HF [9,10]. There are several reasons that may contribute to this. First, despite current recommendations, many hospitals fail to provide adequate education and staff with this designated responsibility [11,12]. The workload of health care professionals is high and is unlikely to decrease, owing to rising health care costs, the current economic downturn, and concomitant cutbacks in staffing [13]. Second, up to 73% of patients with HF have diminished cognitive function [1,14,15], which makes it difficult for them to understand, remember, and apply what they have been taught [16]. Third, many patients with HF have depression [17], which may interfere with their ability to learn and may reduce their motivation to change their behavior [1]. Fourth, even when people have sufficient knowledge, initiating and maintaining behavior changes can be challenging [18,19].

One of the aims of the European Union’s Seventh Framework Programme HeartCycle project was to investigate how telehealth systems could be used to increase adherence to self-care behaviors among patients with HF. At the start of the HeartCycle project in 2008, we analyzed existing efforts to promote self-care among patients with HF and observed that most patients receive self-care education, but get little practical support at home to implement what they have learned. Telehealth systems were focused on the monitoring of vital signs, and, although education on self-care was incorporated in some of them [20], none included coaching patients in changing their behaviors. Researchers in several trials had studied the counseling of patients with HF via telephone interventions performed by trained nurses [21-23]. Although these telephone interventions increased adherence, their main disadvantage was that they were labor-intensive and therefore costly.

In order to have a more cost-effective approach, in the HeartCycle project we developed a novel automated education and coaching (E&C) program and implemented it in a telehealth system. The intention behind this program was not to replace contact with health care professionals, but to enhance the provision of information and to enable behavioral adaptation by means of automated coaching, allowing direct health care consultations to be targeted to more complex needs. We hypothesized that patients can be engaged in HF self-care behaviors by adding a coaching component that uses motivational prompts and feedback to increase their feelings of confidence in and perceived importance of these behaviors, as well as self-regulation tools to let patients set goals and monitor their own progress.

In this paper, we describe the design and use of the HeartCycle E&C program in a multicenter observational trial.

## Methods

### Overview

The HeartCycle E&C program was developed to provide patients with education on HF and associated self-care behaviors and to coach them in adopting these behaviors. In this section, we first summarize the scope, theoretical basis, and delivery infrastructure of the program and then focus on the program itself.

### Program Ingredients

The 2008 guidelines for the diagnosis and treatment of HF issued by the European Society of Cardiology [24] were taken as a starting point to identify the topics and self-care behaviors to be addressed in the program. We decided to offer E&C for daily monitoring of signs and symptoms, physical activity, medication intake, low-salt diet, and fluid restriction. For other topics, such as alcohol consumption and sleep disorders, the program offered education and tips but no explicit coaching. For topics that were considered too sensitive to be dealt with via a telehealth system, such as sexual activity and prognosis, patients were encouraged to seek individual support from their health care team.

Several behavior change theories exist in health psychology, such as the trans-theoretical model of behavior change and the health belief model. Our program is based on the behavior change approach of the Heart Manual. The Heart Manual is the United Kingdom’s leading home-based self-management program for individuals recovering from acute myocardial infarction (AMI). It is facilitated by trained health care professionals, and, through the combination of education, support, and behavioral adaptation, it supports patients after an AMI in many of the same lifestyle behavior changes recommended to patients with HF. The program is based on cognitive behavioral therapy and self-regulation theory, and it employs motivational interviewing. This home-based program is as effective as those administered in cardiac rehabilitation centers [25-28].

To focus the research on self-care rather than on the delivery infrastructure, we chose to reuse the existing Philips Motiva telehealth system, which enables patients to record and transmit vital signs data (eg, weight, blood pressure, pulse) and displays text and videos via a set-top box and TV. The patient uses a remote control to navigate through menus, to select and control educational videos, and to answer multiple-choice questionnaires. The set-top box communicates with a secure back-end server via a secure Internet connection that can be accessed using a PC-based dashboard by authorized HF nurses.

### Program Description

#### Overview

**Figure 1:**
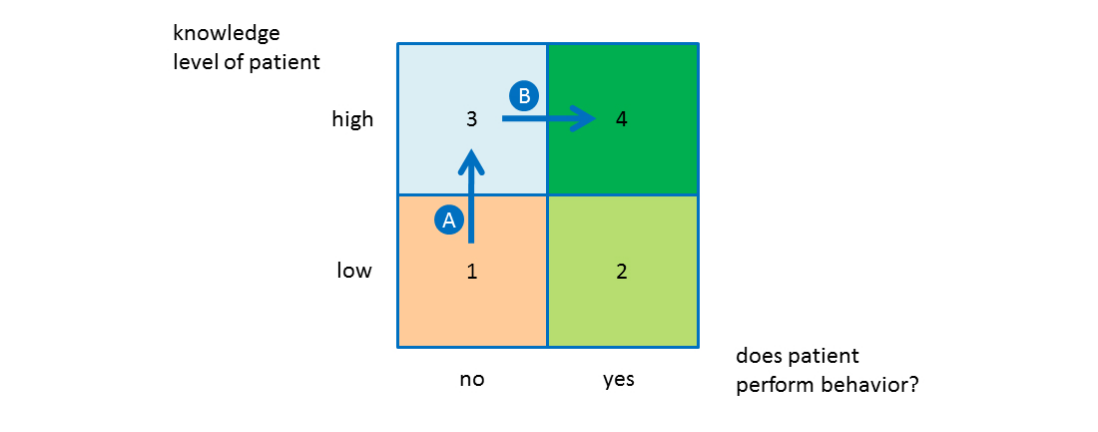
The education and coaching framework. Arrow A denotes education, and arrow B denotes coaching.

The content presented to the patient depends on the patient’s current behavior and knowledge level. An overview of the educational and behavioral possibilities is shown in Figure 1. Ideally, the patient should already have adopted the self-care behavior and know why it is important (ie, the patient is in area 4 for this behavior). If the patient knows what is supposed to be done but does not do it (area 3), the system offers coaching for this behavior (arrow B). If a patient neither knows what to do nor does it (area 1), the system first offers education to increase the patient’s knowledge of this behavior (arrow A) and then provides coaching (arrow B). This mirrors the Heart Manual approach to cardiac rehabilitation, in which the facilitator seeks to first assess the patient’s knowledge and then current behavior, and then provides education and support to target the areas of need.

The remainder of this section describes the steps of the program. Example screenshots are included in Multimedia Appendix 1.

#### Behavior and Knowledge Assessment

For each self-care behavior, the system determines the position of the patient in the matrix shown in Figure 1 by presenting behavior and knowledge questions to the patient. The questions are based on the validated European Heart Failure Self-Care Behaviour Scale [29] and the Dutch Heart Failure Knowledge Scale [30]. However, to assess the patient’s behavior further, we introduced additional questions. For example, we added the statement “I read the label information on food packages to know their salt (sodium) content” to the scale’s statement “I eat a low salt diet.” Furthermore, to make it less likely for the patient to guess the correct answer, we added the answer “I don’t know” to all knowledge questions.

#### Education

Upon completion of the behavior and knowledge assessment, the patient is given access to educational material (videos and textual tips) via the system. The videos and tips are related to the knowledge and behavior topics in the questionnaires. The videos (see Multimedia Appendix 2) contain animations, role models, and expert interviews. If a patient’s score indicates insufficient knowledge of a behavior that he or she is not engaging in, the patient is prompted to watch the corresponding videos before starting with coaching. Hence, the educational content is tailored to the patient’s needs, thus making it more relevant and reducing resistance to the program.

#### Importance and Confidence Assessment

In the motivational interviewing approach to behavior change, an individual’s readiness to change determines the likelihood of attempting to change behaviors [18]. Readiness to change is related to the importance the person places on the change and how confident the person is in making the change. Therefore, for each behavior that a patient is not engaging in, the patient is asked to rate its perceived importance and how confident he or she is in adopting that behavior, and then the patient is given feedback. As low importance is often related to misconceptions [31], the system delivers messages about the most common myths associated with the behavior (eg, “Physical activity is not safe for people with heart failure”) and the corresponding truths (eg, “It is important to be physically active and to rest regularly in between”). For patients with low confidence, which is often related to practical barriers in adopting the behavior [31], the system also gives them information about related solutions and tips. This feedback should address common misconceptions and raise the patient’s motivation to engage with the coaching program.

#### Coaching

**Figure 2:**
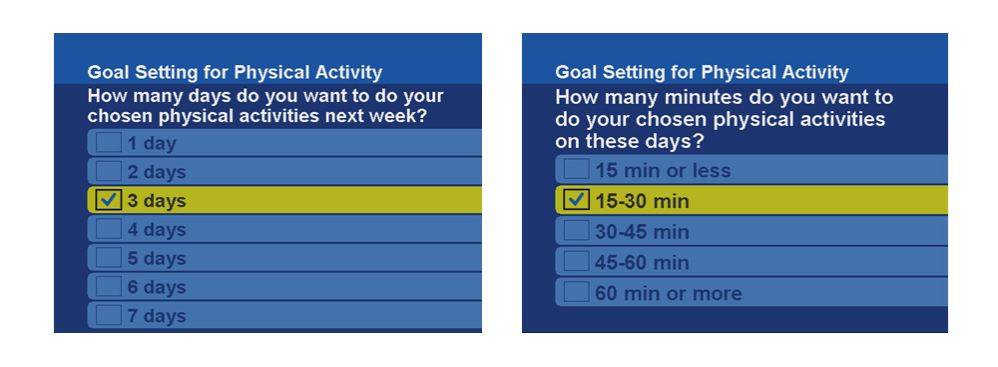
Screenshots from the telehealth system for goal setting for physical activity.

The patient starts the coaching program by setting a personal goal for the following week. The goal depends on the self-care behavior. For example, for physical activity, patients indicate the number of days they want to be active during the following week (eg, 3 d), as well as the number of minutes they will engage in physical activity on those days (eg, 15-30 min/d) (see Figure 2). Every day, the patient enters progress data into the system by means of an on-screen diary. For example, for physical activity, the patient selects the number of minutes (eg, 15-30 min) and rates the effort of the activity (eg, too hard).

At the end of the week, the system provides the patient with feedback on progress. The exact content of the feedback message is tailored to the patient’s goals, progress, and effort rating. For example, if the patient was physically active on fewer days than planned, and the sessions on those days were shorter than planned and too hard, the system gives the patient advice to set a less ambitious goal for the next week.

If the patient continually experiences difficulties in reaching personal goals, the system recommends contacting the HF nurse for further advice and support. Furthermore, it generates an alert in the HF nurse’s dashboard, such that the nurse may decide to call the patient. Hence, although the routine parts of the coaching are automated, the system relies on the nurses for the most challenging parts. This is crucial because, in this way, the nurses can focus on the patients who really need their personal attention, thus allowing an efficient allocation of resources.

#### Adherence

We defined successful adherence to a self-care behavior as reaching personal goals for 2 consecutive weeks. This is a short time frame for achieving sustained behavior change. However, patients were asked to keep a daily on-screen diary, which could have become burdensome over time. We did not want to run the risk that patients would not adopt self-care behaviors because of the progress entry effort, so we chose a 2-week time frame.

When a patient has become adherent, the system acknowledges the patient’s achievement and asks whether the patient wishes to continue to receive coaching. If the patient wishes to continue, the system asks the patient to set new goals for the next week. Otherwise, in 2 months, the system will ask the patient whether this behavior has been maintained. If the patient has relapsed, the system offers the possibility to receive coaching again. Hence, for patients for whom the aforementioned 2-week time frame was too short to achieve sustained behavior change, the system offers a new opportunity to receive coaching.

### Eligibility and Study Design

The evaluation of the E&C program was part of the HeartCycle study. Briefly, HeartCycle was a multicenter observational trial with randomized components in which we investigated the ability of a third-generation telehealth system to enhance the management of patients with HF.

The study had four phases. Phase 1 was an observational period to familiarize the patient with the system, measure the patient’s compliance with monitoring, and assess the patient’s ability to achieve target doses of medications specified in an individual patient care plan, based on European Society of Cardiology guidelines. Care plans were constructed for each patient by an experienced clinician at the site, taking into account factors such as blood pressure, serum potassium concentration, and renal function to modify guideline target doses. Phase 2 comprised 2 randomized cross-over studies. One was for patients with well-controlled symptoms and signs at the end of Phase 1, and compared usual care with a diuretic minimization algorithm. The other included patients with poorly controlled symptoms and signs and compared usual care with a diuretic optimization algorithm. Phase 3 was an observational period in which instructions were given to conduct programmed activities approximately twice per week, such as skipping a medication dose, taking a heavy meal or strong coffee, taking a shower, or engaging in some exercise to see what effect these had on telemonitoring values. Finally, patients entered a longer-term follow-up phase until the last enrolled patient completed Phases 1 to 3 or discontinued monitoring.

Potential participants should have had a recent (<60 d) admission to the hospital for symptoms or signs of HF (either of new onset or recurrent) or were outpatients with persistent New York Heart Association (NYHA) functional class III/IV symptoms despite treatment with diuretics. The inclusion and exclusion criteria are shown in Textboxes 1 and 2. The protocol was reviewed and approved by the ethics committee of each participating center. All patients provided voluntary written informed consent.

It was expected that most patients would be enrolled on cardiology and medical wards during recovery from an episode of worsening HF and that the remainder would be referred from outpatient clinics by research nurses and doctors. Patients were enrolled between January 2012 and February 2013 at three hospital sites within the United Kingdom, Germany, and Spain.

Sample size and power were calculated based on the number of patients required for Phase 2. This calculation was based on the binary endpoint of patient preference for diuretic minimization or optimization compared to standard management using McNemar’s test [32]. All patients participating in the HeartCycle study were offered the E&C program. However, the exact content of the program was tailored to each patient’s specific deficiencies in self-care behavior at the start of the program.

**Inclusion criteria for the HeartCycle study.**

Inclusion criteria:

- A clinical diagnosis of heart failure
  - Cause of heart failure for any reason other than those that are rapidly reversible (see exclusion criteria)
  - May include patients with and without a low left ventricular ejection fraction or with valve disease
- Requiring treatment with at least 40mg/d of furosemide or equivalent (1mg/d of bumetanide or 10mg/d of torasemide)
- Evidence of advanced or unstable disease
  - Admission to hospital for, or complicated by, heart failure currently or within the previous 60 d
  - Outpatients with persistent NYHA III/IV symptoms
- An elevated N-terminal pro-brain natriuretic peptide value within the 3 mo prior to enrollment
  - ≥1000pg/mL if in sinus rhythm, including atrio-biventricular pacing
  - ≥2000pg/mL if not in sinus rhythm

**Exclusion criteria for the HeartCycle study.**

Exclusion criteria:

- Unwilling to comply with the protocol (Patients should be willing and able to take daily measurements at home throughout Phase 2.)
- Rapidly reversible causes of heart failure, such as severe anemia (defined as the need for a blood transfusion), thyrotoxicosis, and/or admission with rapid (>120bpm) atrial fibrillation with good ventricular function
- Inability, in the investigators’ opinion, to operate or comply with the telehealth system, even with available support from caregivers and health care volunteers if available
- Inability to communicate directly or indirectly in the local language (English in the United Kingdom, German in Germany, and Spanish in Spain)
- Persons aged <18 y and vulnerable patient groups, such as those with dementia, psychotic illness, severe intellectual disability, or cognitive dysfunction

### Data Collection and Instruments

The patients received the E&C program via messages, videos, and questionnaires through the telehealth system. The time at which patients viewed these items and their answers to the questionnaires were stored automatically in the telehealth system database. By analyzing a deidentified copy of the database, we gained detailed insight into the patients’ interaction with the system, including the E&C program.

## Results

### Overview

In this section, we first summarize the characteristics of the patients enrolled in the trial. We then show how patients progressed through the assessments and the coaching process. After that, we display the goals of the physical activity and low-salt diet that patients set for themselves. We then present the reasons why several patients did not start the coaching program after completing the behavior and knowledge assessment.

### Patient Characteristics

Of 123 patients enrolled (mean age 66 (12) y, 79% men; see Table 1), 66% were in NYHA class III, indicating that they had marked limitation in physical activity.

**Table 1:** Baseline characteristics of the study population (N=123).

Characteristics		n (%) or mean (SD)
Men		97 (79)
Mean age in years (SD)		66.2 (11.8)
Age >70 y		49 (40)
**BMI, kg/m** ^**2**^		
	Underweight, <18.5	2 (2)
	Normal, 18.5-25.0	37 (30)
	Overweight, 25.0-30.0	45 (37)
	Obese, ≥30.0	39 (32)
**Cardiovascular history**		
	Myocardial infarction	58 (47)
	Revascularization	52 (42)
	Valve surgery	11 (9)
**Comorbidities**		
	Cancer	16 (13)
	Diabetes	53 (43)
**NYHA** ^a^ **functional class/symptoms**		
	NYHA III	81 (66)
	Angina	18 (15)
	Peripheral edema	41 (33)

^a^NYHA: New York Heart Association

### Patient Journeys Through the Coaching Process

Figure 3 shows the observed journeys of patients through the assessments and the coaching process. The model is explained by using physical activity as a specific example of behavior management. The number of patients who made a transition for physical activity at least once is shown next to the arrows. All 123 patients completed the behavior and knowledge assessment. On the basis of this assessment, 50 patients were considered not physically active and received the importance and confidence assessment on physical activity. Thirty-five patients (70%) completed the importance and confidence assessment and started the coaching program. Twenty-eight patients (80%) became adherent, ie, reached their physical activity goals for 2 consecutive weeks.

Eleven of the 28 patients who became adherent chose to stop and 17 continued the coaching program, although 9 of these latter patients subsequently stopped. The remaining 8 patients continued with the coaching program until the end of the study (number not shown in Figure 3), remaining in the state “continued coaching.” Every time these patients achieved their personal physical activity goal for 2 consecutive weeks, they indicated that they wanted to continue the coaching program. One patient indicated this 15 times.

Every 2 months, adherent patients who declined further coaching were sent a questionnaire asking whether they were still adherent (ie, physically active). Of 13 patients, 3 indicated that they had relapsed and that they wanted to receive physical activity coaching again. One patient had relapsed but did not want to try again. Nine patients (number not shown in Figure 3) indicated that they had remained adherent until the end of the study.

For each self-care behavior, Table 2 shows the number of patients who entered a particular state per self-care behavior. For example, for physical activity, 50 (41%) of the 123 patients were nonadherent, 35 (70%) of those 50 patients started coaching, and 28 (80%) of those 35 patients became adherent. Of these latter 28 patients, 11 stopped and 17 continued with coaching after becoming adherent. The numbers for physical activity are also shown in Figure 3.

The distribution of the responses to follow-up questionnaires from patients who stopped coaching after becoming adherent is shown in Table 3. For example, for physical activity, 13 patients received the follow-up questionnaire. Of these 13 patients, 9 reported that they had maintained the behavior, 3 had relapsed and started coaching again, and 1 had relapsed but did not retry coaching.

Because the number of patients who indicated that they did not restrict their fluid intake or did not take their medications as prescribed was very low (6 each; see Table 2), these 2 self-care behaviors are not analyzed or discussed further.

**Table 2:** Number of patients who entered a state per self-care behavior (N=123).

# of patients	Physical activity n (%)	Low-salt diet n (%)	Fluid restriction n (%)	Medication intake n (%)
Were nonadherent	50/123 (40.7)	56/123 (45.5)	6/123 (4.9)	6/123 (4.9)
Started coaching	35/50 (70.0)	47/56 (83.9)	4/6 (66.7)	4/6 (66.7)
Became adherent	28/35 (80.0)	36/47 (76.6)	2/4 (50.0)	4/4 (100.0)
Stopped coaching immediately	11/28 (39.3)	23/36 (63.9)	1/2 (50.0)	1/4 (25.0)
Continued coaching	17/28 (60.7)	13/36 (36.1)	1/2 (50.0)	3/4 (75.0)
Stopped coaching later on	9/17 (52.9)	6/13 (46.2)	0/1 (0.0)	1/3 (33.3)
Continued coaching until study end	8/17 (47.1)	7/13 (53.8)	1/1 (100.0)	2/3 (66.7)

**Table 3:** Patient responses to follow-up questionnaires (N=123).

# of patients	Physical activity	Low-salt diet	Fluid restriction	Medication intake
Received follow-up questionnaire	13	15	1	2
Maintained behavior	9 (69%)	14 (93%)	1 (100%)	2 (100%)
Relapsed and started coaching again	3 (23%)	0 (0%)	0 (0%)	0 (0%)
Relapsed but did not retry coaching	1 (8%)	1 (7%)	0 (0%)	0 (0%)

**Figure 3:**
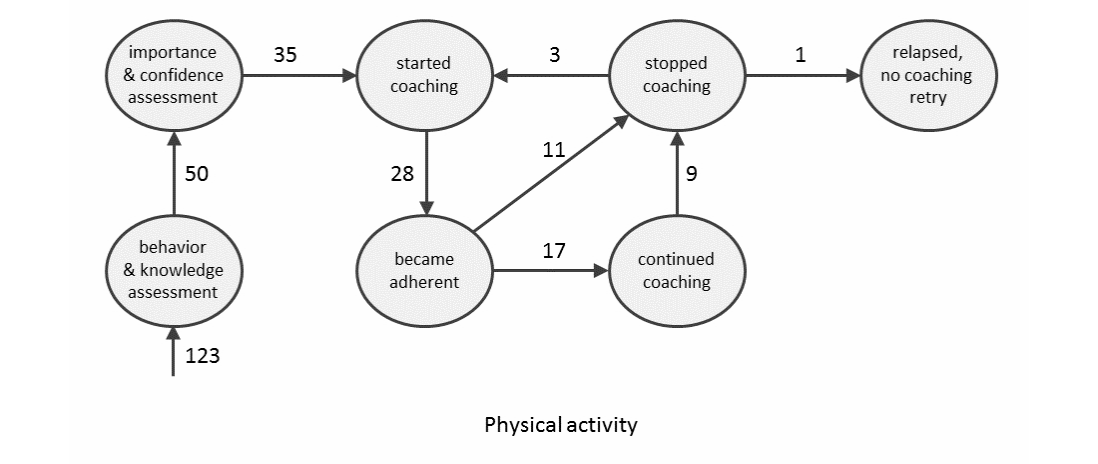
The states (circles) and transitions (arrows) in the coaching process. The numbers at the arrows indicate the number of patients who made this transition for physical activity at least once.

### Goals When Becoming Adherent

We defined a patient as adherent to a self-care behavior if the patient reached a personal goal for 2 consecutive weeks. This section shows the goals that patients set and reached in the second of these 2 weeks.

Table 4 shows the physical activity goals (ie, combination of days and minutes) that patients reached. For example, 3 patients reached a goal consisting of 5 d/wk and 15-30 min/d. In total, 61% (17 of 28) of the patients aimed to be (and reported that they were) active on most (≥4) days of the week, and 75% (21 of 28) of the patients aimed to be (and reported that they were) active ≥15-30 min/d.

Table 5 shows the low-salt diet goals that were reached when patients became adherent. Twelve of the patients set and reported that they reached a goal of 1g/d of sodium, and 56% (20 of 36) set and reported that they reached a goal of ≤2g/d of sodium.

**Table 4:** Distribution of physical activity goals reached when patients became adherent (N=28).

	# of days the patient plans to be active during the week							
# of planned minutes of physical activity on these days	1 day	2 days	3 days	4 days	5 days	6 days	7 days	Subtotal
45-60 min	-	-	-	-	-	-	-	-
30-45 min	-	-	-	-	2	1	1	4
15-30 min	-	1	5	2	3	2	4	17
≤15 min	-	1	4	1	-	-	1	7
Subtotal	-	2	9	3	5	3	6	28

**Table 5:** Distribution of low-salt diet goals reached when patients became adherent (N=36).

Goal	# of patients
≥5 g/d	1
4 g/d	8
3 g/d	7
2 g/d	8
1 g/d	12
Total # of patients	36	

### Reasons for Not Proceeding to Goal Setting

Seventeen patients indicated in the behavior and knowledge assessment that they were not engaging in all self-care behaviors but they did not start the coaching program. Some did not complete the importance and confidence assessment; others did and read the feedback, but did not proceed to goal setting. The reasons for not starting the coaching program are shown in Table 6. Only 1 patient (aged 86 y) did not start coaching because of the program itself, owing to difficulties in understanding the goal-setting component.

Of the 3 patients who were too busy to use the system, 1 was very often absent from home due to employment. Of the 5 patients who had a severe physical or mental impairment, 2 had cancer and felt that they could not cope with coaching on lifestyle activities, 2 had severe HF, and 1 (aged 38 y) had difficulty coping with the HF diagnosis. Four patients who had difficulties in understanding the system had cognitive limitations and needed help from a family member to operate the system. Most technical problems were related to a slow Internet connection.

**Table 6:** Reasons for not starting coaching program.

Reason	n
Death	1
Patient too busy	3
Severe physical or mental impairment	5
Lack of motivation	1
Difficulties in understanding the system	4
Difficulties in understanding goal setting	1
Unknown	1
Technical problems with the system	4

## Discussion

### Principal Findings

This study shows that our automated coaching program was effective for most patients who started coaching. In regard to the physical activity and low-salt diet components, about 80% of the patients who engaged in them achieved their personal goals within 2 weeks.

The mean age (66±12 y) of the study participants was relatively low, and the study population was dominated by men (79%). The mean age of patients admitted to the hospital with HF in Western Europe is about 78 years. However, the mean age at admission for men and for patients with HF and a low left ventricular ejection fraction is about 5 years younger [33]. Older patients are more likely to have cognitive dysfunction. In addition, older patients may be less willing to participate in research, thus accounting for the low numbers of elderly patients in most studies of HF.

The physical activity goals that the patients set for themselves are consistent with the most recent HF guidelines [34]. Most patients who became adherent to physical activity goals were active on ≥4 d/wk for ≥15-30 min/d. Considering that 66% of the patients in our study had NYHA III HF, this result seems positive. Patients with HF are known to have low adherence to physical activity recommendations, so additional strategies to improve adherence are required. Home-based initiatives, such as our automated coaching program, are a promising strategy for overcoming several barriers and for improving and maintaining physical function and fitness [35,36].

Our program was also successful in increasing adherence to a low-salt diet. We included this self-care behavior in our program, taking as a starting point the guidelines for the diagnosis and treatment of HF as published by the European Society of Cardiology in 2008 [24]. The newer guidelines from 2012 question the effectiveness of salt restriction in patients with HF [34], and some people argue that accepting thirst and tasteless food is very difficult. However, in a recent study, researchers showed that individualized salt and fluid restrictions can improve signs and symptoms of HF with no negative effects on thirst, appetite, or quality of life in patients with moderate to severe HF and previous signs of fluid retention [37].

Seventeen patients who indicated in the behavior and knowledge assessment that they were not engaging in all self-care behaviors did not start the coaching program. This was sometimes due to slow Internet connections, but more often was related to patient factors such as disease severity or comorbidity that rendered coaching inappropriate.

Our study also shows that there was a large variation in the percentage of patients who continued coaching after they became adherent, depending on the behavior. For physical activity, 61% (17 of 28) of patients continued coaching after becoming adherent, but only 36% (13 of 36) of patients adherent to a low-salt diet continued coaching. This may reflect the fact that once knowledge was obtained and applied, the patients knew what to do and did not feel the need for more coaching. Alternatively, patients may feel that some goals are less important or desirable than others and thus decide not to continue to adopt that change in behavior. The follow-up questionnaires showed that 93% of patients said they maintained a low-salt diet, suggesting the first of these two explanations. The percentage of patients who indicated that they maintained the behavior when they received the follow-up questionnaire also varied based on the self-care behavior. For physical activity and low-salt diet, these data were 69% (9 of 13 patients) and 93% (14 of 15 patients), respectively.

Many patients continued to use the on-screen diary when they reached their goals, suggesting either that they felt some sort of benefit, possibly as motivational support for behavior change, or that they at least found it easy to adopt. For these patients, the benefits of the automated coaching program clearly outweighed the fact that they had to manually enter their progress in a daily on-screen diary. More than 40% of the patients who continued with coaching after they became adherent continued with it until the end of the study.

One of the limitations of this study is that the program is based on patient self-reporting. In particular, whether a patient received coaching for a particular self-care behavior depended on the patient’s self-reported adherence to the behavior at the start of the program. We could have integrated objective tools such as accelerometers or pedometers for physical activity, 24-hour urinary excretion (with well-known difficulties and inaccuracies) for dietary salt intake, and medication dispensers, but resources did not allow us to do so. Similarly to other studies [38], virtually all patients in our study self-reported that they were adherent to their medication prescriptions. As a consequence, we could not test the effectiveness of the medication adherence promotion part of the program.

A strength of this automated program is that it goes one step further than the simple education and information delivery model by providing coaching (ie, explicit help in adopting self-care behaviors) in the home context. Furthermore, the program is innovative in the provision of automated motivational feedback according to the levels of importance and confidence expressed by the patient. This feedback should address common patient misconceptions and increase patient motivation to engage with the coaching program. Finally, the program takes into account the dynamics of health-related behavior changes by explicitly addressing relapse. Patients who relapse are offered the opportunity to repeat the coaching program.

Since we started the development of our program in 2008, surprisingly few initiatives using automated self-care coaching for patients with HF have been begun. To the best of our knowledge, only one study (CHF-CePPORT) has aims to establish and evaluate an e-platform for behavioral counseling and education to facilitate long-term adherence to self-care among patients with HF [39]. The investigators in the TEHAF study also addressed adherence to HF self-care [40,41]; however, although their telehealth system offers personalized advice, it does not use a goal-setting approach as ours does. Researchers in another study investigated whether mobile phones could be used for vital signs measurements and symptom monitoring by patients with HF [42,43]. Although the results of that study were very positive, the system did not offer e-counseling on self-care behaviors. Hence, as far as we know, the HeartCycle study is the first in which an advanced automated coaching approach to promote self-care among patients with HF has been developed and tested.

Adherence to most self-care behaviors is poor among patients with HF [9,10]. Various initiatives have been undertaken to remedy this problem, including in-hospital education, nurse-led disease management programs, and education via digital media. In the present study, we explored an alternative approach and achieved promising results. Nevertheless, more research is needed to understand the extent to which the various components of the program contributed to the result, to investigate how self-reporting can be replaced by more objective tools, and to achieve even higher adoption percentages.

### Conclusions

The HeartCycle E&C program helped patients with HF to adopt recommended self-care behaviors. Automated coaching worked very well for most patients who started coaching, and many patients who achieved their goals continued to use the coaching program.

The patients who did not engage in the automated coaching program seemed to be inappropriate candidates for this approach. If appropriate for these patients, increased face-to-face contact with an HF nurse may be required to support self-management.

## Multimedia Appendix 1

Example screenshots illustrating the steps in the program.

## Multimedia Appendix 2

A fragment from the video on physical activity.
