# Supplementary material for: Design and Usage of the HeartCycle Education and Coaching Program for Patients With Heart Failure
Source: JMIR Res Protoc. 2014 Dec 11;3(4):e72. doi: 10.2196/resprot.3411 (PMC4275507; doi:10.2196/resprot.3411)
Supplement: Supplementary file 1 [file resprot_v3i4e72_app1.pdf]

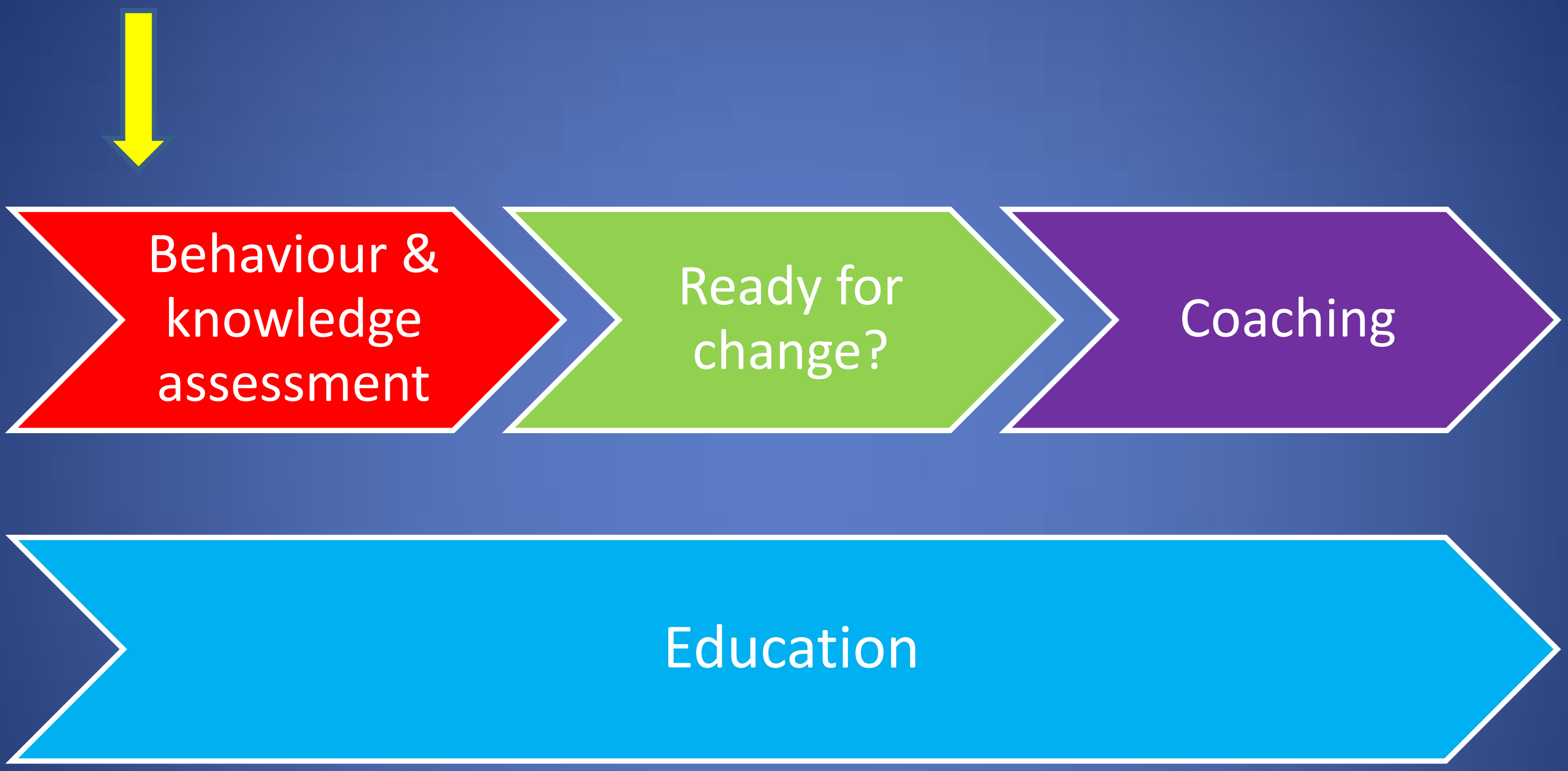
Behaviour & knowledge assessment
Ready for change?
Coaching
Education

HeartCycle

Questions on Salt Consumption

We would like you to answer some questions on salt consumption.

Please answer the questions honestly, as this will help us to provide information which suits your own specific needs.

To continue, press ☑

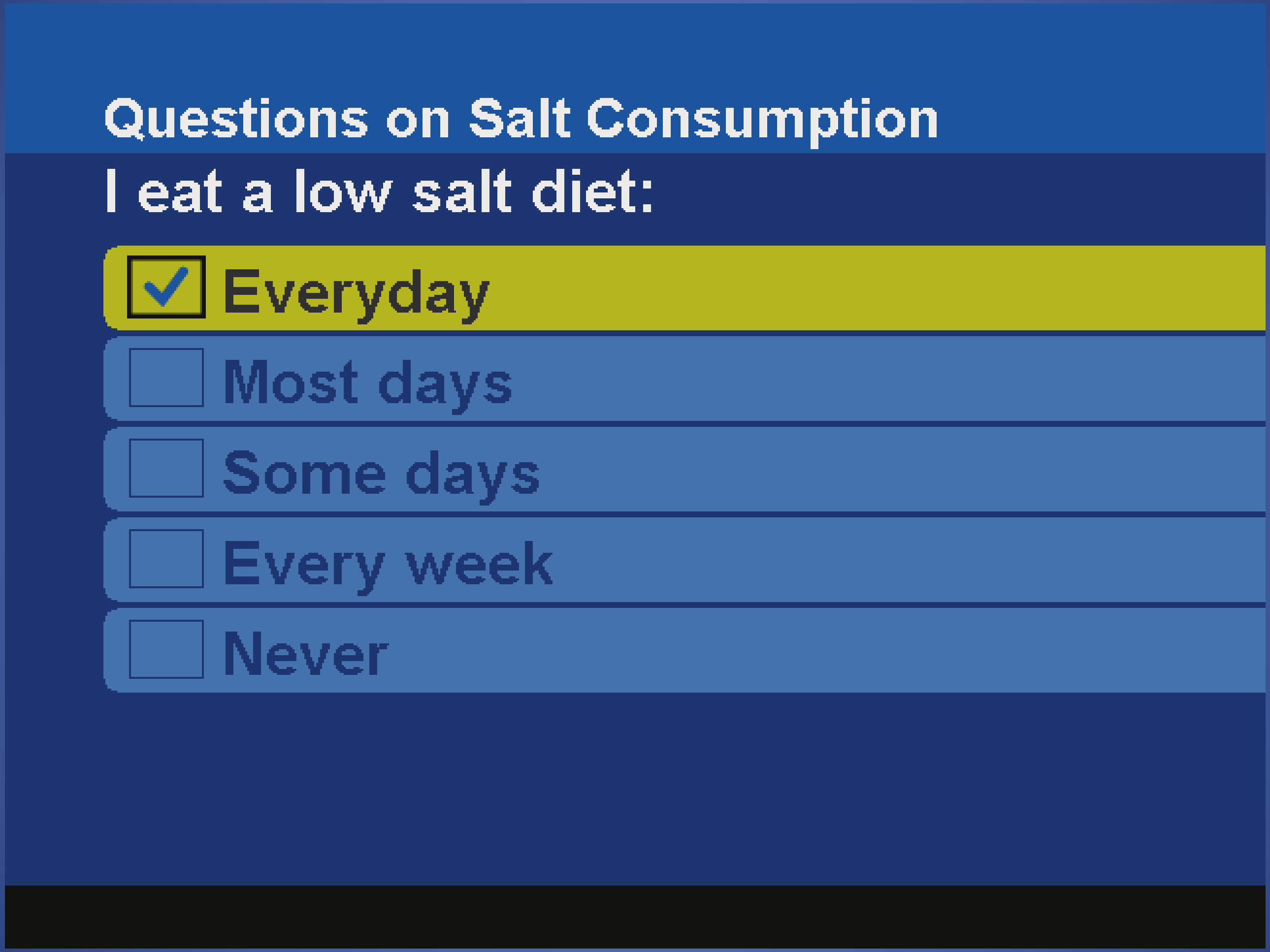
Questions on Salt Consumption
I eat a low salt diet:
Everyday
Most days
Some days
Every week
Never

Questions on Salt Consumption

I read the label information on food packages to know their salt (sodium) content:

- [ ] Always
- [ ] Often
- [ ] Sometimes
- [x] Hardly ever
- [ ] Never

Questions on Salt Consumption

It is very good that you eat a low salt diet. In this way you are helping to keep your heart condition under control.

Some food items, such as canned soups, contain "hidden" salt. It is therefore important to read food labels too.

To continue, press ☑

HeartCycle

Questions on Salt Consumption

We would like to ask you one more question on low salt diet.

If you are unsure of the answer, simply select the option 'I don't know'.

To continue, press [✓]

Questions on Salt Consumption

Why should someone with heart failure follow a low salt diet?

- [ ] Salt increases fluid retention
- [x] Salt interferes with my medication
- [ ] Salt makes me feel more tired
- [ ] I don’t know

Questions on Salt Consumption

Actually, salt can upset the body's fluid balance causing it to retain more fluid. This means that your heart will have to work harder.

To continue, press ☑

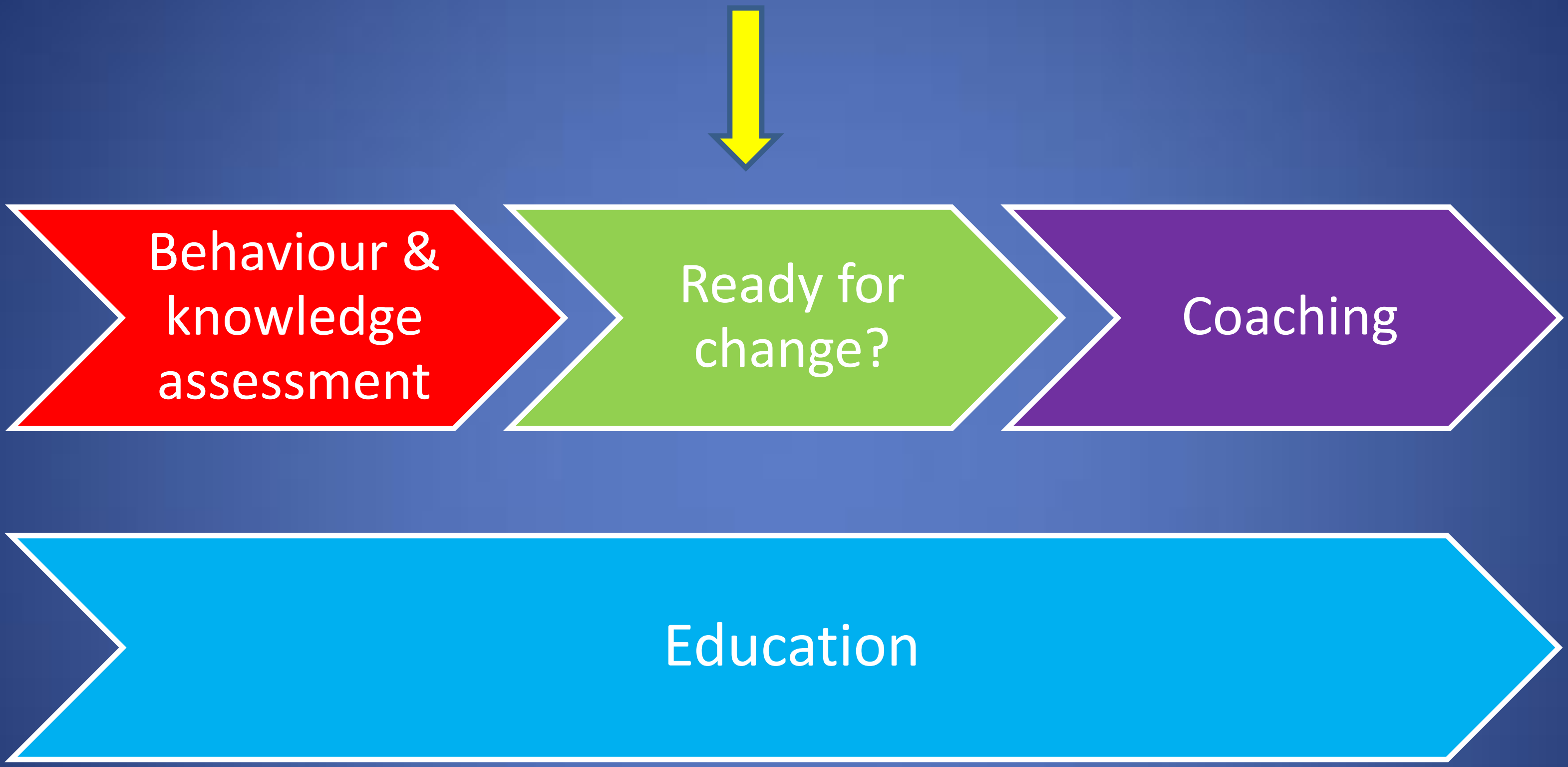
Behaviour & knowledge assessment
Ready for change?
Coaching
Education

Physical Activity - Importance and Confidence

We would like you to answer 2 questions about physical activity.

Please answer the questions honestly, as this will help us to provide information which suits your own specific needs.

To continue, press ☑

Physical Activity - Importance and Confidence

How important do you think it is for you to be physically active?

- ☐ 1 - not important at all
- ☐ 2 - not so important
- ☐ 3 - neutral
- ☑ 4 - important
- ☐ 5 -very important

Physical Activity - Importance and Confidence

How confident are you that you can lead a physically active life?

- [ ] 1 - not confident at all
- [x] 2 - not so confident
- [ ] 3 - neutral
- [ ] 4 - confident
- [ ] 5 -very confident

Review of Physical Activity

Myth 2:

"There is no point in being physically active. It's too late if you have heart failure."

To continue, press ☑

Review of Physical Activity

Truth — There are many benefits to remaining active, even if you've already got heart failure.

It can strengthen your heart, help reduce risk factors and generally make you feel better.

To continue, press ☑

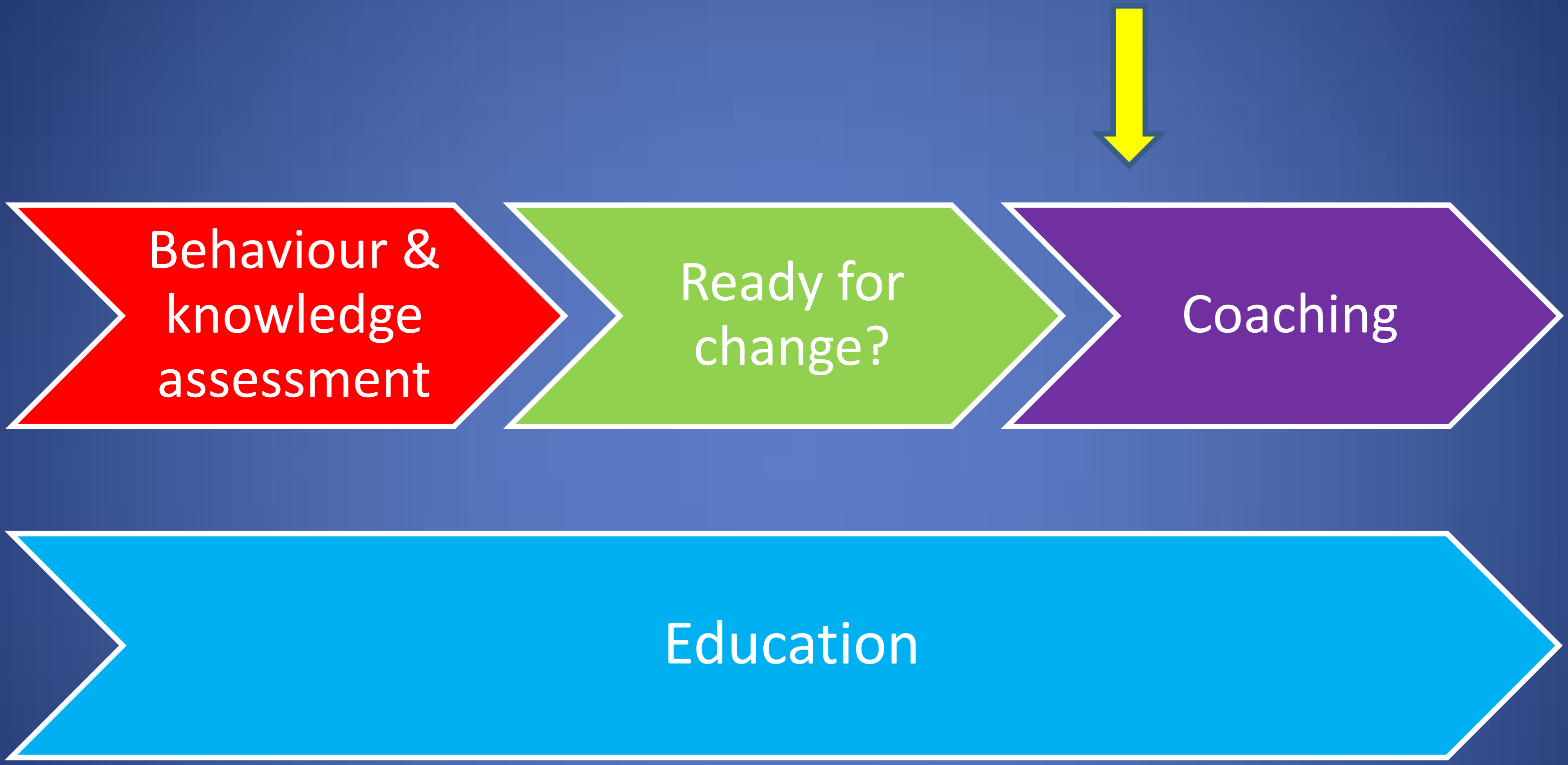
Behaviour & knowledge assessment
Ready for change?
Coaching
Education

Goal Setting for Physical Activity

We will now help you to set goals for physical activity.

Think of activities you would like to do next week (e.g. walking, cycling, dancing, swimming,...).

To continue, press ☑

Goal Setting for Physical Activity

Please enter the target number of days per week and target number of minutes per day which would be fairly easy for you to do these activities.

To continue, press ☑

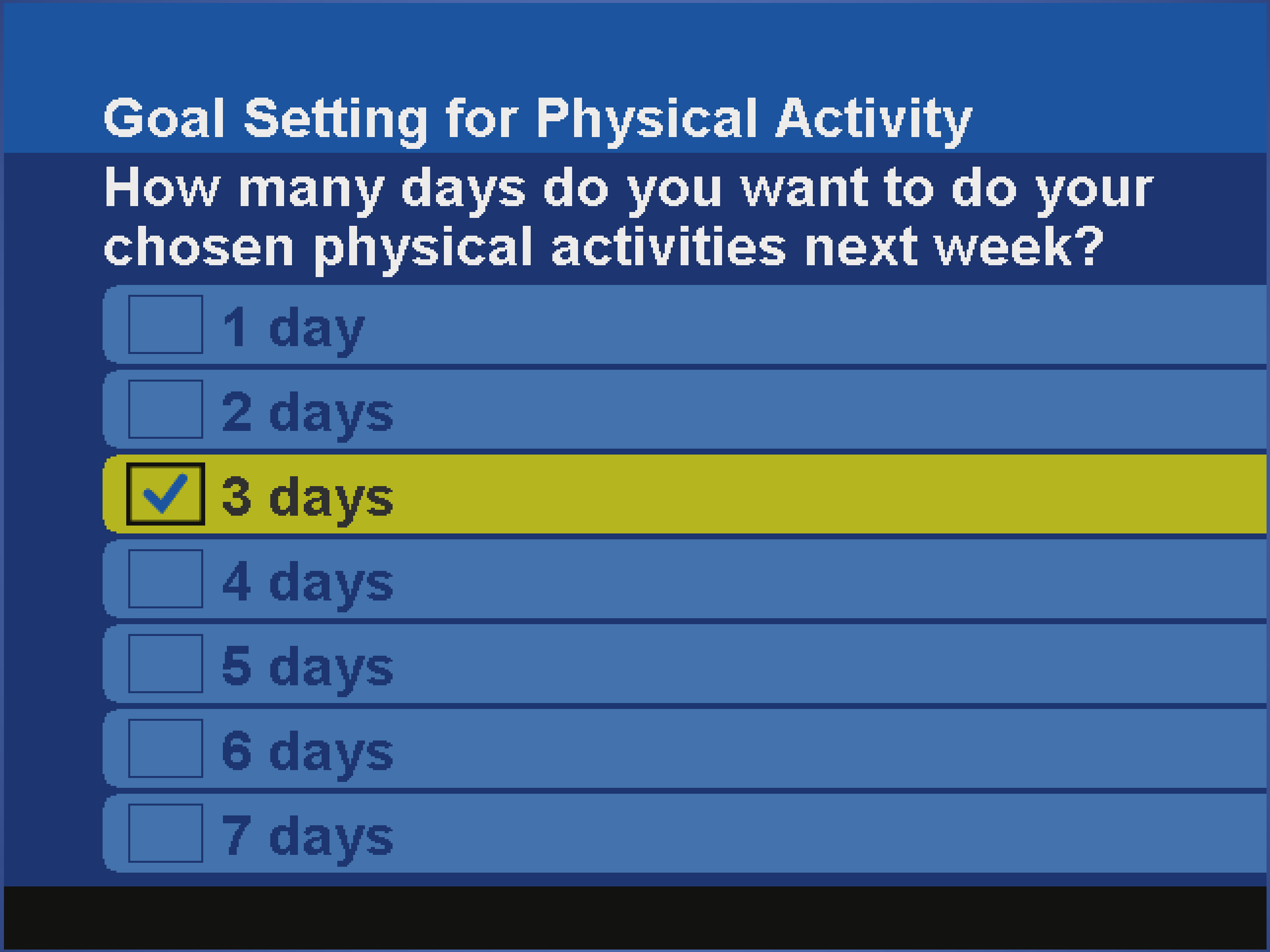
Goal Setting for Physical Activity
How many days do you want to do your chosen physical activities next week?
1 day
2 days
3 days
4 days
5 days
6 days
7 days

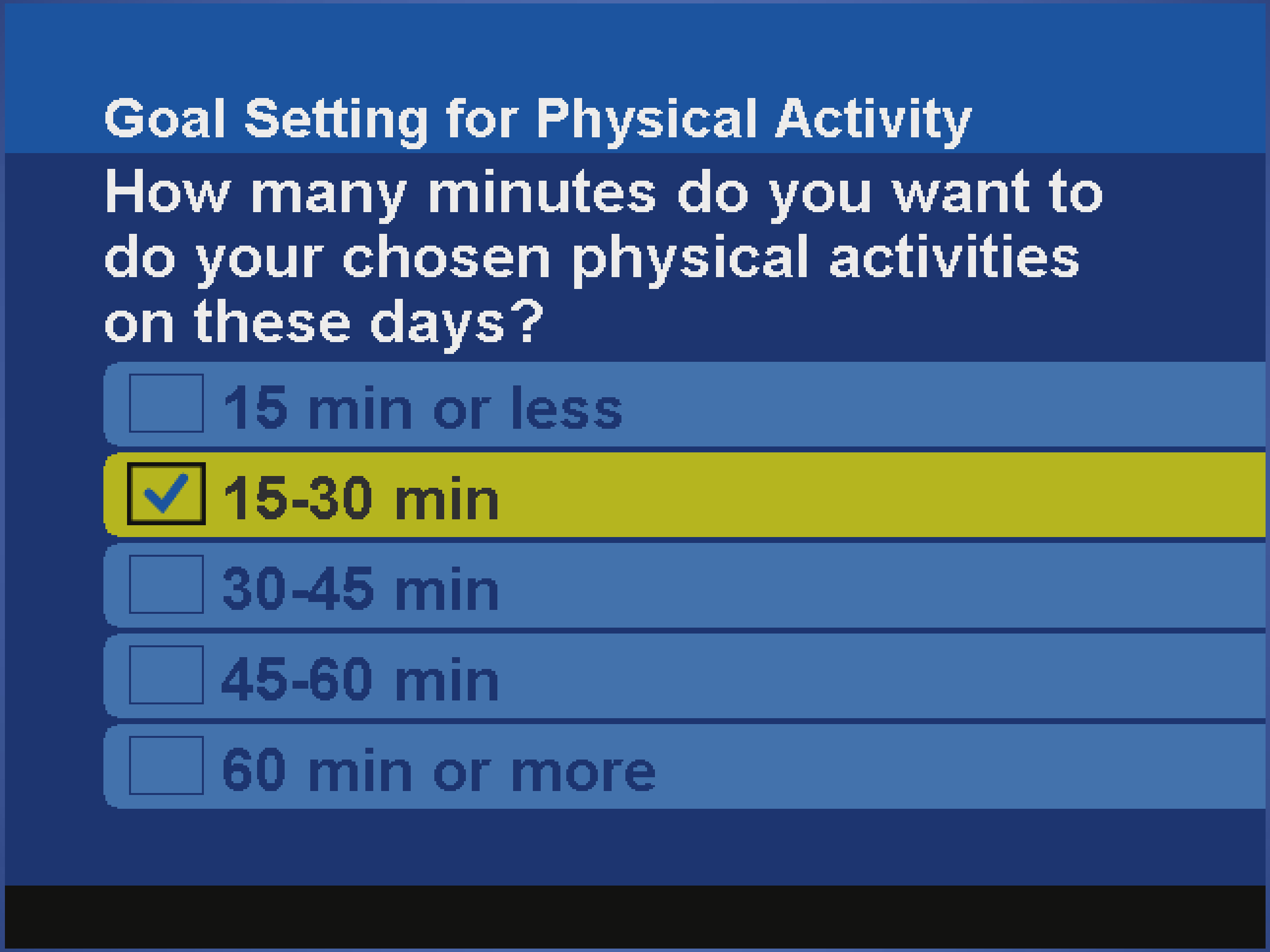
Goal Setting for Physical Activity
How many minutes do you want to do your chosen physical activities on these days?
15 min or less
15-30 min
30-45 min
45-60 min
60 min or more

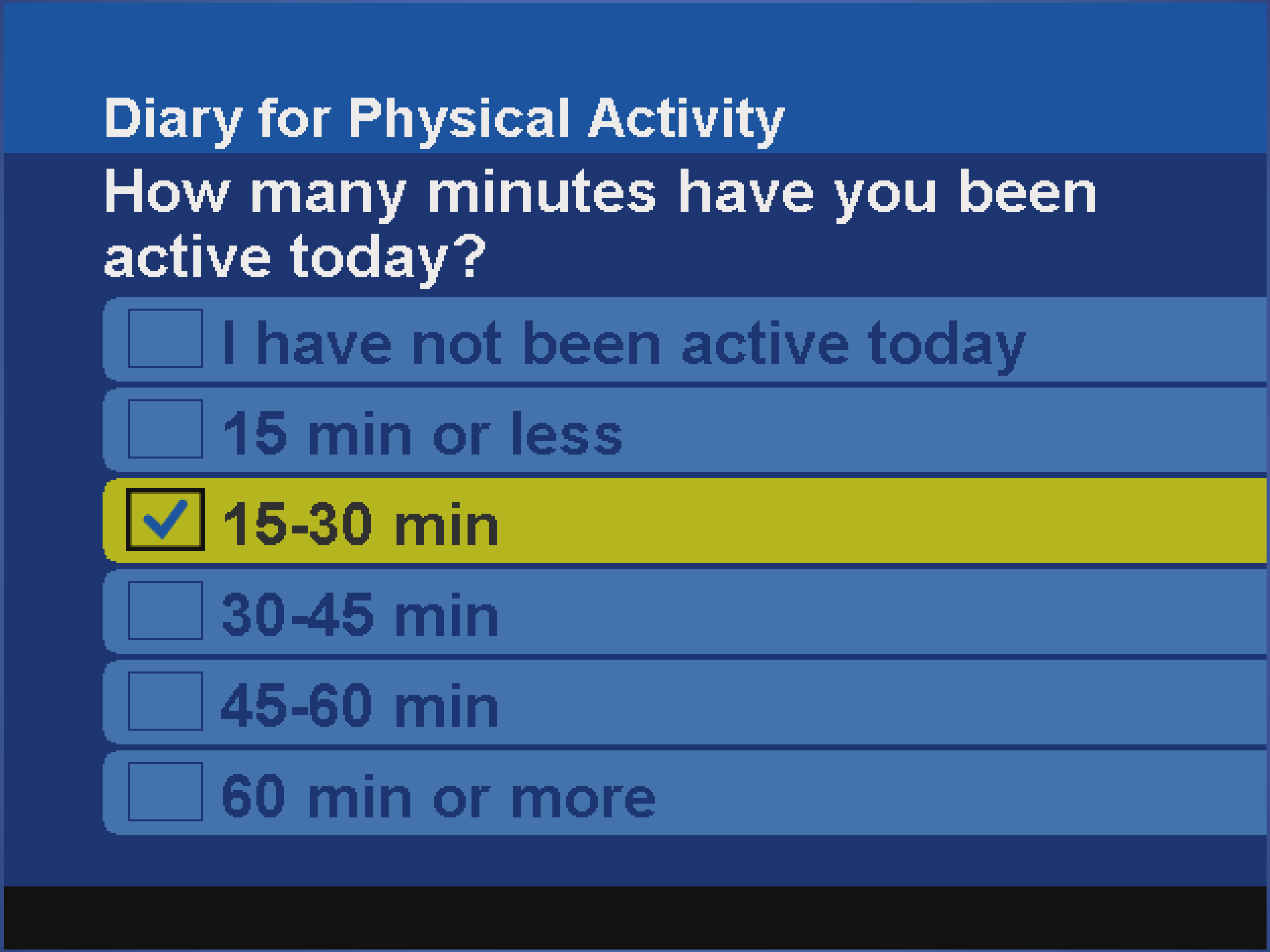
Diary for Physical Activity
How many minutes have you been active today?
I have not been active today
15 min or less
15-30 min
30-45 min
45-60 min
60 min or more

HeartCycle

Diary for Physical Activity

Please rate the effort of this activity.

- [ ] Too easy
- [x] Fairly easy
- [ ] Just right
- [ ] Fairly hard
- [ ] Too hard

Feedback on Physical Activity

Well done! Last week you reached your target number of days and minutes.

Based on your effort rating, we conclude that the activity was ‘easy’ for you.

Please now set your goal for this week such that it becomes 'just right'.

To continue, press ☑
